# Evaluation of a Rapid Dipstick (Crystal VC) for the Diagnosis of Cholera in Zanzibar and a Comparison with Previous Studies

**DOI:** 10.1371/journal.pone.0036930

**Published:** 2012-05-25

**Authors:** Benedikt Ley, Ahmed M. Khatib, Kamala Thriemer, Lorenz von Seidlein, Jacqueline Deen, Asish Mukhopadyay, Na-Yoon Chang, Ramadhan Hashim, Wolfgang Schmied, Clara J-L. Busch, Rita Reyburn, Thomas Wierzba, John D. Clemens, Harald Wilfing, Godwin Enwere, Theresa Aguado, Mohammad S. Jiddawi, David Sack, Said M. Ali

**Affiliations:** 1 Translational Research Division, International Vaccine Institute, Seoul, Korea; 2 Biocenter, University of Vienna, Vienna, Austria; 3 Ministry of Health and Social Welfare, Zanzibar, Tanzania; 4 Global Health Division, Menzies School of Health Research, Casuarina, Australia; 5 National Institute of Cholera and Enteric Diseases, Kolkata, India; 6 Institute of Molecular Biotechnology, Vienna, Austria; 7 World Health Organization, Geneva, Switzerland; 8 John Hopkins Bloomberg School of Public Health, Baltimore, Maryland, United States of America; 9 Public Health Laboratory (Pemba) – Ivo de Carneri, Chake Chake, Tanzania; University of Otago, New Zealand

## Abstract

**Background:**

The gold standard for the diagnosis of cholera is stool culture, but this requires laboratory facilities and takes at least 24 hours. A rapid diagnostic test (RDT) that can be used by minimally trained staff at treatment centers could potentially improve the reporting and management of cholera outbreaks.

**Methods:**

We evaluated the Crystal VC™ RDT under field conditions in Zanzibar in 2009. Patients presenting to treatment centers with watery diarrhea provided a stool sample for rapid diagnostic testing. Results were compared to stool culture performed in a reference laboratory. We assessed the overall performance of the RDT and evaluated whether previous intake of antibiotics, intravenous fluids, location of testing, and skill level of the technician affected the RDT results.

**Results:**

We included stool samples from 624 patients. Compared to culture, the overall sensitivity of the RDT was 93.1% (95%CI: 88.7 to 96.2%), specificity was 49.2% (95%CI: 44.3 to 54.1%), the positive predictive value was 47.0% (95%CI: 42.1 to 52.0%) and the negative predictive value was 93.6% (95%CI: 89.6 to 96.5%). The overall false positivity rate was 50.8% (213/419); fieldworkers frequently misread very faint test lines as positive.

**Conclusion:**

The observed sensitivity of the Crystal VC RDT evaluated was similar compared to earlier versions, while specificity was poorer. The current version of the RDT could potentially be used as a screening tool in the field. Because of the high proportion of false positive results when field workers test stool specimens, positive results will need to be confirmed with stool culture.

## Introduction

Cholera remains a very common and potentially lethal disease in Asia and Africa. Globally, more than 220,000 cases were reported to the World Health Organization (WHO) in 2009 [1], however the true number of cases, including unreported cases, is likely to be much higher – perhaps 3–5 million cases/year [2]. Cholera occurs mainly in areas with poor infrastructure and limited access to clean water. The etiologic organisms, Vibrio cholerae O1 and O139, are highly transmissible and can cause explosive outbreaks. While many of those affected experience only mild symptoms, some suffer from severe disease characterized by profuse diarrhea, electrolyte imbalance, coma and death if prompt rehydration is not provided [3], [4]. Cholera cases have been reported from Zanzibar since 1978 with regular outbreaks documented since then [5], [6].

The gold standard for laboratory confirmation of cholera is stool culture [7]. This is a routine procedure but requires laboratory infrastructure including trained staff. A single stool culture costs approximately 4 USD/case [8] and requires about 24 to 72 hours and transport to the closest sufficiently equipped laboratory, which may create additional costs. Furthermore, microbiologic facilities are often not available in locations where cholera occurs. A rapid diagnostic test (RDT) that is simple, easy to use and interpret, can be stored without refrigeration and is reasonably priced so that it can be deployed widely would be useful for the early confirmation of cholera outbreaks. Ideally, the RDT should be highly sensitive so as not to miss the diagnosis of cases and be sufficiently specific when used under actual field conditions [9]. Cholera confirmation would enable immediate implementation of control measures such as reactive vaccination [6], as well as more accurate reporting of the burden of the disease.

A cholera RDT based on the detection of lipopolysaccharide (LPS) using gold particles was developed by the Institute Pasteur (IP). The RDT is a lateral flow immunochromatographic test for the qualitative determination of lipopolysaccharide antigen of both *Vibrio cholerae* O1 and O139 serogroups from stool specimens using monoclonal antibodies specific to *V. cholerae* O1 and O139 LPS. Through a licensure agreement, the RDT is now being produced by Span Diagnostics (Surat, India) under the trade name Crystal VC™ at a price of 19.00 USD/test kit (10 test strips). The test kit is stable at temperatures between 4°C to 30°C, and test strips are packed in waterproof pouches, allowing storage under high humidity conditions. Previous evaluations have been performed on the prototype and commercial versions of the RDT [10]–[15]. The primary objective of this study is to validate the current version of the Crystal VC™ RDT when performed by health workers in first-level treatment centers in Zanzibar. We also sought to assess if the RDT results were affected by the skill level of the reader and previous intake of antibiotics or intravenous fluids.

## Methods

### Ethics

The study was conducted according to the principles expressed in the Declaration of Helsinki. Written informed consent was obtained from all participants. The Zanzibar Research Council Ethics Committee, the Institutional Review Board of the International Vaccine Institute, Seoul, Korea, and the Research Ethics Review Committee of the World Health Organization, Geneva, Switzerland approved this project.

### Study site

**Figure 1:**
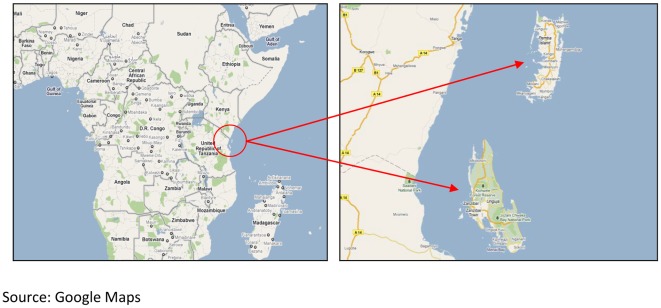
Study site.

The archipelago of Zanzibar lies about 50 kilometers east of mainland Tanzania and consists of two main islands, Unguja and Pemba, as well as smaller islets (Figure 1). In 2009, Zanzibar had a population of about 1.22 million [16]. Stool samples were collected at cholera treatment camps that were set up during outbreaks on the two main islands, Unguja and Pemba, in 2009. Treatment of patients was provided according to national guidelines.

### Study procedures

Acute watery diarrhea was defined as a minimum of three liquid, non-bloody, stools within 24 hours. Prior to presentation, no further inclusion/exclusion criteria were applied. Patients presenting with acute watery diarrhea were requested to provide a stool sample in a disposable plastic container. A swab was inserted into the stool sample and used to inoculate a tube of pre-packaged Cary Blair medium (EIKEN, Japan) for transport to the laboratory. About 200 µl of stool from each sample was used for dipstick testing on site. A case report form (CRF) was completed to record frequency of bowel movement over the previous 24 hours, antibiotics received, and fluid management (intravenous (IV) or oral rehydration solution (ORS)) provided at the health center. Bulk stool from a subset of patients attending a camp close to one of the participating laboratories was transported to the lab for additional testing on the same day as described below.

#### Dipstick test

*The RDT was sto*red at room temperature and performed according to the package insert. Liquid stool was collected in a disposable plastic container. Approximately 200 µl (4 drops) of stool were transferred with a disposable pipette to a disposable test tube provided with the kit. One drop of dilution buffer was added. The dipstick was inserted into the diluted stool and results were read within 15–20 minutes. The appearance of two bands on the dipstick, one control and one test, indicated that the stool sample was positive for *V. cholerae*. Th*e appearanc*e of only the control band indicated a negative sample. The non-appearance of the control band indicated a procedural error. Stool samples were tested under field conditions in the cholera treatment centers and in the laboratory as described below. Dipstick results were recorded on the CRF, whereas laboratory results were recorded in separate laboratory forms.

- Performed under field conditions. A local health worker in each cholera treatment camp performed the RDT after training and a copy of the English test kit manual containing illustrations on test procedure and interpretation had been provided. Training consisted of a theoretical session using a Power Point Presentation containing information on test procedures and schematic pictures of positive and negative test results based on the package insert. This was followed by a practical session during which the test was performed a number of times. All field workers where visited frequently in the field to ensure correct handling of the test. All local health workers had completed at least primary education and delivered basic medical services to attending diarrheal patients. Fieldworkers performed the test outdoors in daylight.
- Performed under laboratory conditions. In order to assess the potential influence of environmental and light conditions, laboratory technicians were asked to repeat the test on bulk stool collected at the camps and to read the result independently. Stool culture results were not yet available at the time of the performance of the test and laboratory technicians were blinded as to the results of the RDT performed in the field, as well as the clinical picture of the patient. Two laboratory technicians performed the RDT after receiving training similar to the field workers and had received a copy of the test kit manual. The laboratory technicians performed the test indoors using electric light sources. All participating technicians had a diploma in laboratory sciences, which requires a minimum of one year of education, and had a minimum of three years working experience.

#### Stool culture

*Upon arrival in* the laboratory, samples from the Cary Blair media were streaked out on Thiosulphate Citrate Bile Sucrose Agar (TCBS; EIKEN, Japan), inoculated in alkaline peptone water (APW) and incubated at 37°C for 12–24 hours. If samples arrived as bulk stool, the samples were diluted in APW. An aliquot was streaked out on TCBS and the samples in APW and on TCBS were incubated for 12–24 hours at 37°C. If no growth on TCBS was detected after incubation, an aliquot of the sample in APW was streaked out on TCBS and incubated again. If yellow colonies indicative of *V. cholerae* wer*e detected* on TCBS, motility indole ornithine agar (MIO) and triple sugar iron agar (TSI) were inoculated with colonies from TCBS and incubated for 18 hours at 37°C. In addition, a colony from TCBS was sub-cultivated on gelatin agar for later serological confirmation and incubated at 37°C overnight. If colonies indicative of *V. cholerae* wer*e observed* on TSI and MIO after incubation, colonies from gelatin agar were tested for agglutination reactions with O1 polyvalent, O1 Inaba, O1 Ogawa and O139 antiserum (Beckton Dickinson, USA) as described elsewhere [17]. *V. cholerae* str*ains were t*ransported to the National Institute of Cholera and Enteric Diseases in Kolkata, India where identification of the isolates was confirmed.

### Definitions, data management and analysis

The CRF and laboratory results of each patient were computerized and linked using unique study identification numbers. The primary endpoint was the assessment of the performance of the RDT (done in the field) using microbiological stool culture result as the gold standard for comparison. Sensitivity (true-positive or TP rate) was defined as the probability that patients with laboratory-confirmed cholera had a positive RDT. Specificity (true-negative or TN rate) was the probability that patients with no laboratory-confirmed cholera had a negative RDT. The positive predictive value (PPV) was the probability that patients with a positive RDT had *V.cholerae* isolated from stool culture. The negative predictive value (NPV) was the probability that patients with a negative RDT had no *V.cholerae* isolated from a stool culture. The false positivity or FP rate ( = FP/[FP+TN] or 1−specificity) was the proportion of stool samples with no *V. cholerae* isolated on culture but showed a positive RDT result. The false negativity or FN rate ( = FN/[TP+FN] or 1−sensitivity) was the proportion of stool samples with *V. cholerae* isolated on culture but showed a negative RDT result.

We performed sub-group analyses by island (Pemba or Unguja), by previous recent intake versus non-intake of antibiotics and by receipt of intravenous fluids following previously published studies showing differences in RDT performance [13]. We defined recent intake of antibiotics as receipt of any oral or parenteral antimicrobial for the current illness prior to the collection of a stool sample. We classified fluid management at the treatment center as oral rehydration solution (ORS) or intravenous fluids (IVF) with or without ORS. To assess whether test validity was related to skill level of the reader and location of the testing, we compared the performance of the RDT when done in the field versus in the laboratory on a subset of samples.

Comparison of unpaired samples was done using chi-square test; comparison of paired samples was done using McNemars test. Confidence intervals were calculated using exact method. Level of agreement was calculated using Cohen's Kappa test for unweighted proportions. Calculations were done using Stata, version 10 (StataCorp, College Station, TX, USA). Performance indicators were calculated using Excel 2010 (Microsoft, WA, USA).

## Results

**Figure 2:**
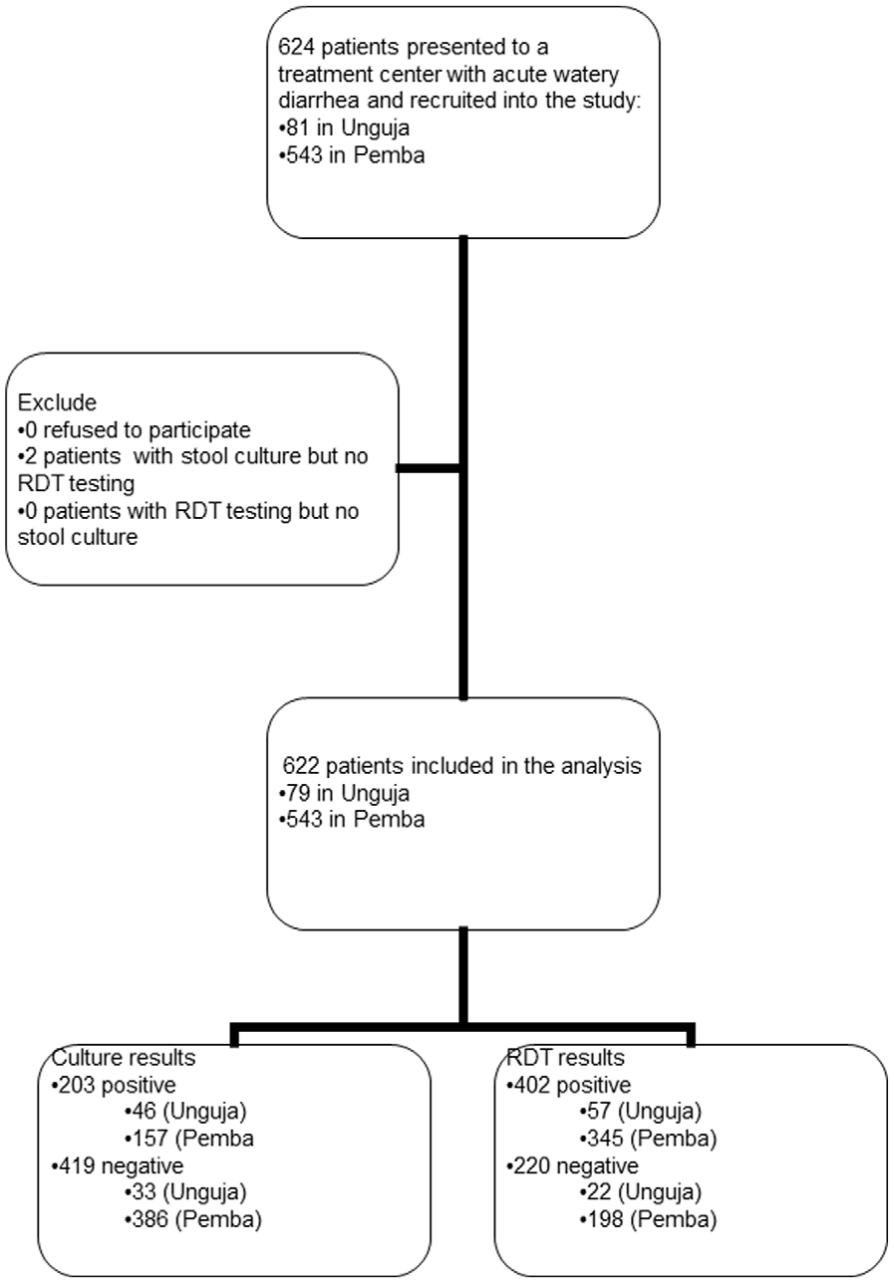
Flow of study participants.

There were 624 patients who presented to a cholera treatment camp with acute watery diarrhea and were recruited into the study: 81 in Unguja and 543 in Pemba. We excluded 2 samples sent for culture but on which no RDT was done. A total of 622 stool samples were included in the analysis, 79 (13%) from Unguja and 543 (87%) from Pemba residents (Figure 2).

### Performance of the RDT in the field

Of the 622 stool samples, 203 (32.6%) yielded *V. cholerae* O1. No *V. cholerae* O139 was isolated. Using culture results as the gold standard, we calculated the sensitivity, specificity, PPV, and NPV of the RDT performed in the field for the diagnosis of cholera (Table 1). Overall sensitivity was 93.1% (95%CI: 88.7 to 96.2%), specificity was 49.2% (95%CI: 44.3 to 54.1%), the positive predictive value (PPV) was 47.0% (95%CI: 42.1 to 52.0%) and the negative predictive value (NPV) was 93.6% (95%CI: 89.6 to 96.5%). The overall false positivity rate was 50.8% (213/419).

**Table 1:** Performance of the cholera rapid diagnostic test, Pemba and Unguja, Zanzibar.

	Sensitivity% (95%CI*) (TP/(TP+FN))	Specificity% (95%CI*) (TN/(TN+FP))	PPV% (95%CI*) (TP/(TP+FP))	NPV% (95%CI*) (TN/(TN+FN))	No. pos./total (%) with *V. cholera* isolated on stool culture
**Total (n = 622)**	93.1 (88.7–96.2) (189/203)	49.2 (44.3–54.1) (206/419)	47.0 (42.1–52.0) (189/402)	93.6 (89.6–96.5) (206/220)	203/622 (32.6)
**Unguja (n = 79)**	89.1 (76.4–96.4) (41/46)	51.5 (33.5–69.2) (17/33)	71.9 (58.5–83.0) (41/57)	77.3 (54.6–92.2) (17/22)	46/79 (58.2)
**Pemba (n = 543)**	94.3 (89.4–97.4) (148/157)	49.0 (43.9–54.1) (189/386)	42.9 (37.6–48.3) (148/345)	95.5 (91.5–97.9) (189/198)	157/543 (28.9)
**p**	**0.82**	**0.87**	**0.02**	**0.53**	**<0.01**

* using exact method.

### Sub-group analyses of performance of the RDT in the field

We evaluated the RDT performance by island (Table 1). *V. cholerae* was isolated from 46/79 or 58.2% of stool samples from Unguja compared to a significantly lower proportion of 157/543 or 28.9%, from Pemba (p<0.01). No significant differences in sensitivity, specificity and NPV of the RDT were observed between Unguja and Pemba, as well as between each island with the overall results (all p>0.05). However, we found a significant difference in PPV between Unguja and Pemba (71.9, 95%CI: 58.5–83.0 versus 42.9, 95%CI: 37.6–48.3; p = 0.02).

We compared the RDT performance by fluid management of patients: Rehydration treatment at the cholera camp was recorded for 592/622 (95.2%) participants (Table 2). Only 15.2% (32/210) of participants who received oral rehydration had a positive stool culture, compared with 43.2% (165/382) of those who received IV fluids (p<0.01). There were no statistically significant differences in sensitivity, specificity and NPV of RDT performance by fluids received (all p>0.05). However we again found a significant difference in PPV among those who received oral compared to IV rehydration (26.8%, 95%CI: 18.9 to 36.0% versus 55.2%, 95%CI: 49.2 to 61.2%; p<0.01). To evaluate whether the provided fluid treatment biased the field workers' interpretation of the RDT results, we compared the false positivity rate by fluid management. The false positivity rate was 46.1% among those who were orally rehydrated and 57.1% among those who were intravenously rehydrated (p = 0.22).

**Table 2:** Stratified analysis of the performance of cholera dipstick test according to fluid management (oral rehydration or intravenous fluids) in 592 patients and recent antibiotic intake (yes or no) in 576 patients.

	Sensitivity (95%CI)* (TP/(TP+FN))	Specificity (95%CI)* (TN/(TN+FP))	PPV (95%CI)* (TP/(TP+FP))	NPV (95%CI)* (TN/(TN+FN))	No. pos./total (%) with *V. cholerae* isolated on stool culture
**Fluid management (n = 592)**					
**Oral rehydration n = 210**	93.8 (79.2–99.2) (30/32)	53.9 (46.3–61.4) (96/178)	26.8 (18.9–36.0) (30/112)	98.0 (92.8–99.8) (96/98)	32**/210 (15.2)
**Intravenous rehydrationn = 382**	92.7 (87.6–96.1) (153/165)	42.9 (36.2–49.7) (93/217)	55.2 (49.2–61.2) (153/277)	88.6 (80.9–94.0) (93/105)	165**/382 (43.2)
**p**	0.97	0.19	<0.01	0.62	<0.01
**Recent antibiotic (AB) intake (n = 576)**					
**AB received n = 58**	93.8 (69.7–99.8) (15/16)	59.5 (43.3–74.4) (25/42)	46.9 (29.1–65.3) (15/32)	96.2 (80.4–99.9) (25/26)	16/58 (27.6)
**No AB received n = 519**	92.8 (87.8–96.2) (155/167)	49.3 (43.9–54.7) (173/351)	46.5 (41.1–52.1) (155/333)	93.5 (86.1–94.7) (173/190)	167/518 (32.2)
**p**	0.98	0.48	0.98	0.86	0.60

* using exact method.

** in 6/203 culture positive cases no rehydration treatment was provided.

Information on prior antibiotic treatment was recorded for 576/622 (92.6%) participants. The percentage with a positive stool culture was 27.6% among those who had received antibiotics and 32.2% among those who had not (p = 0.60). We assessed whether previous antibiotic treatment affected the RDT performance (Table 2). Sensitivity, specificity, PPV and NPV did not vary significantly among recipients and non-recipients of antibiotics. The false positivity rate was 40.5% among those who had received antibiotics and 50.7% among those who had not (p = 0.45).

### Comparison of the performance of the RDT in the field versus in the laboratory

We compared the performance of RDT on a subset of 67/79 (84.8%) stool samples from Unguja tested both in the field and in the laboratory (Table 3). In this subset, 40/67 (59.7%) samples yielded *V. cholerae* on culture. There was no statistically significant difference in the sensitivity, specificity PPV and NPV of the RDT's performance (all p>0.05). The false positivity rate of the RDT was 45.4% in the field and 25.9% in the laboratory (Cohen's kappa 0.8).

**Table 3:** Comparison of the field and laboratory performance of the cholera dipstick test.

(n = 67)	Sensitivity% (95%CI*) (TP/TP+FN)	Specificity% (95%CI*) (TN/TN+FP)	PPV% (95%CI*) (TP/TP+FP)	NPV% (95%CI*) (TN/TN+FN)	No. (%) with *V. cholera* isolated on stool culture**
**Field**	90.0 (76.3–97.2) (36/40)	55.6 (35.3–74.5) (15/27)	75.0 (60.4–86.4) (36/48)	78.9 (54.4–93.9) (15/19)	40 (59.7)
**Laboratory**	87.5 (73.2–95.8) (35/40)	74.1 (53.7–88.9) (20/27)	83.3 (68.6–93.0) (35/42)	80.0 (59.3–93.2) (20/25)	40 (59.7)
**P** ***	0.931	0.510	0.740	0.977	

* using exact method.

** rapid diagnostic testing was done both in the field and in the laboratory on specimens from the same stool samples.

*** using McNemars test.

### Operational characteristics

The test procedure, excluding sample collection, requires 20–25 minutes. The test kit manual provides clear instructions, and handling of the test was considered simple by field workers. Field workers found it easy to distinguish between valid and non-valid test results, based on the appearance of a control line. However, very faint positive test lines were interpreted as a positive result, but could not be confirmed by culture.

## Discussion

We found an overall high sensitivity (93.1%) of the current version of the cholera RDT consistent with previous reports but a much poorer specificity (49.2%). Earlier studies were performed using a prototype dipstick – developed by the Institute Pasteur – and earlier versions of the commercial kit. Studies using the prototype versions of the RDT reported sensitivities in the range of 93% to 99% and specificities of 67%–97% [12]–[15]], whereas more recent reports on earlier versions of the commercial kit showed sensitivities of 92–97% and specificities of 71–76% [10], [11]. These studies not only varied by the RDT version used but also by the methodology and qualifications of the study personnel performing the RDT (Table 4). There were also variations in the test procedure such as addition or non-addition of a buffer solution to the sample. In some studies, a 4-hour incubation step in alkaline peptone water was added. Overall, the prototype and precursor commercial kits performed better than the current version tested.

**Table 4:** Validation studies of the Institut Pasteur prototype and Crystal VC™ test for diagnosis of *Vibrio cholerae*(O1 samples only considered).

RDT	Institut Pasteur prototype				Crystal VC™ (Span Diagnostics, India)		
Authors	Nato, et.al. [14]	Bhuiyan, et.al. [13]	Wang, et.al. [12]	Kalluri, et.al. [11]	Harris, et.al. [9]	Mukherjee et al. [10]	This Paper
**Journal, Year**	CDLI, 2003	JCM, 2003	BMC Inf. Dis., 2006	TM&IH, 2006	TM&IH, 2009	JJID, 2010	This Journal
**Samples from**	Madagascar	Bangladesh	Mozambique	Bangladesh	Guinea-Bissau	India	Zanzibar/Tanzania
**Gold standard; type of stool samples used**	Culture of frozen stool or rapid cultures of stool collected on filter paper	Culture of rectal swab in C-B media (and multiplex PCR when RDT+/culture -)	Culture of rectal swabs in C-B media and bulk stool	Culture of bulk stool	PCR of swab in C-B media	Culture of bulk stool	Culture of rectal swab in C-B media
**Type of stool samples for RDT testing**	frozen stool/rapid cultures of stool collected on filter paper	Rectal swab incubated in APW at 37°C for 4 h	Rectal swab incubated in APW at 37°C/4 h and fresh, bulk stool	Fresh, bulk stool	Fresh, bulk stool	Fresh, bulk stool	Fresh, bulk stool
**RDT done by**	?	?	Trained technician at field laboratory	Trained technician at ICDDR,B and trained field ‘paramedics’	Individuals with graduate-level laboratory technical training	Post graduate investigator	**Field worker at cholera treatment center (total)** *Field worker at cholera treatment center (subset) Lab. tech. (subset)*
**No of samples**	140	134	219 rectal swabs in APW 172 bulk stool	304	101	212	**622 (total)** *67(subset)*
**No (%) positive for VC O1 by gold standard**	65 (46%)	68 (51%)	138 (35%)	116 (38%)	65 (64%)	72 (34%)	**203 (33%) (total)** *40 (60%) (subset)*
**Sensitivity**	98.5%	96%	97% rectal swabs in APW 93% bulk stool	94% lab tech. 93% field ‘paramedics’	97%	92%	**93% (total)** *88% lab tech (subset) 90% field worker (subset)*
**Specificity**	96%	92%	97% rectal swabs in APW 77% bulk stool	76% lab tech. 67% by field ‘paramedics	71–76%;	73%	**49% (total)** *74% lab tech (subset) 56% field worker (subset)*
**PPV**	95.6%	93%	94% rectal swabs in APW 74% bulk stool	70% lab tech. 63% field ‘paramedics	87–89%	64%	**47% (total)** *83% lab tech (subset) 75% field worker (subset)*
**NPV**	99%	95%	?	95% lab tech. 94% field ‘paramedics	92–93%	95%	**94% (total)** *80% lab tech (subset) 79% field worker (subset)*

The poor specificity of the current version of the commercial kit was associated with an overall false positive rate of 50.8%. The RDT's false positivity rate when the RDT was read in the field was 44% versus 26% when done by laboratory technicians; possibly faint test lines on the dipsticks were over-read by field workers as positive. We hypothesized whether patient characteristics (fluid management or receipt of antibiotics) biased the field workers' interpretation of the RDT results. However, we found no significant differences in false positivity in these sub-group analyses. More likely the fieldworkers over-interpreted faint test lines which could be recognized in daylight but not in the indoor laboratory setting.

Previously, Kalluri et al. assessed the impact of the reader's qualification on the performance of the prototype test [12]. Laboratory technicians with several years of working experience as well as field workers with at least a college degree but no laboratory experience were asked to perform the test on 304 stool samples. The reported RDT sensitivities of 94% and 93% when done by laboratory technicians and field workers, respectively, were similar, but RDT specificity was higher when performed by the technicians (76% versus 67%) [12]. Harris et al. report a sensitivity of 97% and a specificity of 71–76%, when staff with graduate-level laboratory training performed the test in Guinea Bissau [10]. Mukherjee et al. reported a similar sensitivity and specificity (92% and 73%, respectively) when the test was done by graduate-level staff during a surveillance study at a hospital in Kolkata [11].

In contrast to Wang et al., we did not find a higher sensitivity of the RDT when testing stool samples from patients receiving IVF compared to samples from patients who did not receive IVF [13]. However, we noted that the PPV in this study varied according to the proportion of culture-positive specimens. It has been argued that PPV is the most important measure of a clinical diagnostic method since it represents the proportion of patients with positive test results that are correctly diagnosed [18]. The PPV is not intrinsic to the test; it is affected by prevalence of the disease. For example, the PPV was 55% for samples from patients given IVF (43% of whom had *V. cholerae* isolated) while it was 27% for samples from patients managed with ORS (15% of whom had *V. cholerae* isolated). The PPV was 71.9% for the Unguja sub-sample with 58% cholera confirmation while for the Pemba sub-sample it was 43% with 29% cholera confirmation. In outbreak settings, when a large proportion of patients presenting with acute watery diarrhea have cholera, a positive RDT result would have a good predictive value. In other situations (e.g. areas with seasonal cholera but also high rates of diarrheal diseases from other pathogens), the RDT may be less useful.

Our study has several limitations. Firstly, a large sample from 622 study participants was available for the overall evaluation, but only 67 stool samples were used for sub-analyses. Secondly, while confirmation of *V. cholerae* isolates was performed at a reference laboratory, culture-negative stool samples were not validated further. In particular, we did not perform PCR testing on our RDT-positive, culture-negative samples. Bhuiyan, et al. [14] analyzed five stool samples collected in Bangladesh by multiplex PCR that were O1 dipstick positive but culture-negative and found that all five were negative by PCR, indicating that the five dipstick-positive results were false positives. This is reassuring but does not entirely exclude the possibility of false negativity by stool culture. Thirdly, Alam et al. pointed out that the dipstick may detect non-culturable forms of *V. cholerae* that have transformed into a coccoid form due to unfavorable intra-host conditions, such as antibiotic treatment prior to testing [7]. We tried to assess the influence antibiotic treatment prior to sample collection may have had on our results but found no significant difference (p>0.05) in the false positive rates among participants who had taken antibiotics prior to sample collection and those who had not. However, further research is needed to rule out the possibility that the RDT may detect *V. cholerae* antigen in some specimens which are culture negative.

### Conclusion

We found that field workers in this study who had basic general education but were not familiar with laboratory work experienced difficulties in interpreting the RDT performed in the cholera camps. If the RDT is to be deployed more widely, more extensive and repeated training may be required to improve the current RDT's specificity. The test cannot replace stool culture and due to the high number of false positive results observed is not suitable to trigger an outbreak response in a resource poor setting. However the test may be potentially used as a screening tool. During cholera outbreaks, especially when several samples test positive, the test has an enhanced predictive value. Further research is needed to evaluate the accuracy of the RDT with specimens which have been incubated in APW for 4 to 6 hours prior to testing in the RDT since this procedure should dilute out the materials in stool samples which are causing the false positive results while amplifying the antigen signal from the *V. cholerae*.

## References

[1] World Health Organization (2011). Number of Cholera Cases.. http://www.who.int/gho/epidemic_diseases/cholera/situation_trends_cases/en/index.html.

[2] World Health Organization (2011). Cholera: Fact sheet No. 107.. http://www.who.int/mediacentre/factsheets/fs107/en/.

[3] Center for Disease Control (2010). Cholera.. http://www.cdc.gov/cholera/disease.html.

[4] Global Task Force on Cholera Control (2004). Case management: treatment.. Cholera Outbreak - Assessing The Outbreak Response And Improving Preparedness.

[5] Chaignat C-L (2006). Cholera outbreak response in Pemba, 2006 And Pertinence of using Oral Cholera Vaccines for Control.. http://www.who.int/cholera/technical/CholeraZanzibarReviewJuly2006.pdf.

[6] Reyburn R, Deen JL, Grais RF, Bhattacharya SK, Sur D (2011). The case for Reactive Mass Oral Cholera Vaccinations.. PLoS Negl Trop Dis.

[7] Alam M, Hasan NA, Sultana M, Nair GB, Sadique A (2010). Diagnostic Limitations to Accurate Diagnosis of Cholera.. Journal of Clinical Microbiology.

[8] Riewpaiboon A, Intrapakan K, Phoungkatesunthorn S (2008). Predicting Treatment Cost for Bacterial Diarrhoea at a Regional Hospital in Thailand.. J Health Popul Nutr.

[9] The TDR Diagnostics Evaluation Expert Panel (2010). Evaluation of diagnostic tests for infectious diseases: general principles.. Nature Reviews – Microbiology.

[10] Harris JR, Cavallaro EC, de Nobrega AA, dos S Barrado JC, Bopp C (2009). Field Evaluation of Crystal VC Rapid Dipstick test for cholera during a cholera outbreak in Guinea - Bissau.. Tropical Medicine and International Health.

[11] Mukherjee P, Ghosh S, Ramamurthy T, Bhattacharya MK, Nandy RK (2010). Evaluation of a Rapid Immunochromatographic Dipstick Kit for Diagnosis of Cholera Emphasizes Its Outbreak Utility.. Jpn J Infect Dis.

[12] Kalluri P, Naheed A, Rahman S, Ansaruzzaman M, Faruque AS (2006). Evaluation of three rapid diagnostic tests for cholera: does the skill level of the technician matter?. Tropical Medicine and International Health.

[13] Wang X-Y, Ansaruzzaman M, Vaz R, Mondlane C, Lucas ME (2006). Field evaluation of a rapid immunochromatograpgic dipstick test for the diagnosis of cholera in a high-risk population.. BMC Infectious Diseases.

[14] Bhuiyan NA, Qadri F, Faruque AS, Malek MA, Salam MA (2003). Use of Diptsicks for Rapid Diagnosis of Cholera Caused by Vibrio cholerae O1 and O139 from Rectal Swabs.. Journal of Clinical Microbiology.

[15] Nato F, Boutonnier A, Rajerison M, Grosjean P, Dartevelle S (2003). One-Step Immunochromatographic Dipstick Tests for Rapid Detection of Vibrio cholerae O1 and O139 in Stool Samples.. Clinical And Diagnostic Laboratory Immunology.

[16] National Bureau of Statistics, Ministry of Planning, Economy and Empowerment (2006). Analytical Report, Volume X, p2.. http://www.nbs.go.tz/pdf/2002popcensus.pdf.

[17] Bopp C, Ries AA, Wells JG, Koplan JP, Hughes JM (1999). Laboratory Methods for the Diagnosis of Epidemic Dysentery and Cholera.. http://www.cdc.gov/ncidod/dbmd/diseaseinfo/cholera/complete.pdf.

[18] Altman DG, Bland JM (1994). Diagnostic tests 2: Predictive values.. BMJ.

